# Inflammatory Myofibroblastic Tumor of Common Bile Duct in a Girl

**DOI:** 10.21699/ajcr.v7i4.445

**Published:** 2016-09-01

**Authors:** Aureen D’Cunha, Susan Jehangir, Reju Thomas

**Affiliations:** Department of Pediatric Surgery, Christian Medical College, Vellore,INDIA

**Keywords:** Inflammatory myofibroblastic tumor, Pseudotumor, Common bile duct

## Abstract

Inflammatory myofibroblastic tumor (IMT) is a rare, low grade malignant lesion which can occur anywhere in the body. In children it is usually found in the visceral soft tissues with a potential for local invasion and recurrence, and rarely distant metastasis. We report the diagnostic dilemma faced in the management of a 12-year old girl who presented with obstructive jaundice with a mass lesion at the distal end of the common bile duct. She underwent a tumor resection with a bilio-enteric bypass followed by a course of oral steroids and celecoxib.

## INTRODUCTION

IMTs grouped under inflammatory pseudo-tumours are true neoplasms with rarely reported metastasis.[1] IMTs of the biliary tree are extremely rare in children.[2,3] Herein we report a 12-year old female with obstructive jaundice in whom a diagnosis of IMT was finally made.

## CASE REPORT

A 12-year-old girl presented with right upper quadrant abdominal pain associated with progressive jaundice and fever for two months. She was passing high coloured urine, clay coloured stools and had pruritus since the onset of symptoms. She also had loss of appetite and weight. There was no history of abdominal distension and fever. On admission she was noted to be cachectic and deeply icteric with a tender hepatomegaly. There were no stigmata of chronic liver disease, free fluid or other palpable masses in abdomen.

Liver functions were deranged and suggestive of an obstructive pathology, with a total bilirubin of 13.86 mg/dL (range: 0.5-1.0 mg/dL), direct bilirubin of 12.1 mg/dL and alkaline phosphatase of 943 U/L (range: 40-125 U/L). Bleeding parameters and serum albumin were in the normal range. Viral serology was negative. Abdominal ultrasonogram (US) showed central intrahepatic biliary dilatation, a dilated gall bladder and upper common bile duct (CBD) to 9.3mm with gall bladder sludge. There was no evidence of cholelithiasis. MRCP confirmed the US findings. The distal CBD was not visualized. Computed tomogram (CT) showed central intrahepatic biliary radical dilatation with an abrupt cut-off at the region of mid and distal CBD by an ill-defined attenuated soft tissue lesion (Fig.1). The pancreatic head was bulky with a normal main pancreatic duct. ERCP was attempted, but failed due to obscured anatomy of the ampulla of Vater. A percutaneous trans-hepatic biliary drainage (PTBD) was established in view of the persistent jaundice and poor general condition of the child. A dye study through the PTBD confirmed the cut-off at mid CBD level. A working diagnosis of a long segment stricture of the distal CBD due to a mass lesion of unknown etiology was made. The differential diagnoses considered were tuberculosis, lymphoma, primary sclerosing cholangitis, connective tissue disorders and lastly, a cholangiocarcinoma. The work up for tuberculosis and connective tissue disorders was negative and the bile culture did not grow organisms. The lesion was not amenable for endoscopic ultrasound biopsy, image guided biopsy or cytology.

**Figure:**
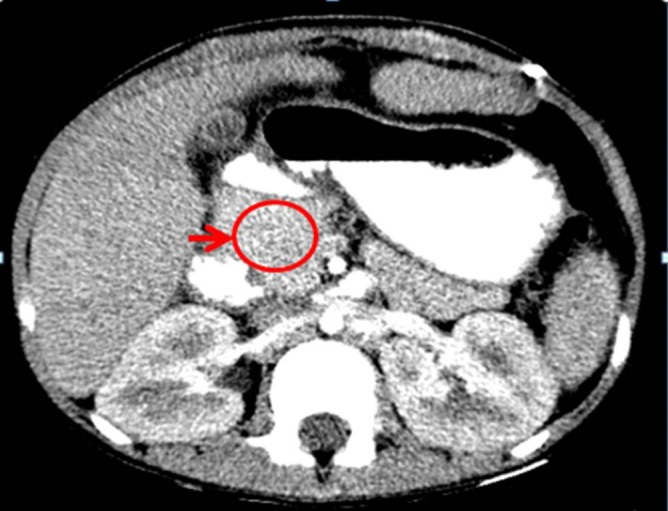
Figure 1:CT scan showing mass lesion in the vicinity of distal CBD and pancreatic head.

At surgery, a firm mass was found at the distal CBD and pancreatic head measuring approximately 4cm x4cm with multiple periportal lymph nodes. Histopathology on the frozen section was inconclusive. Considering the rarity of a cholangiocarcinoma in the paediatric population and a higher probability of lymphoma, it was decided to perform multiple biopsies and plan for definitive treatment based on the final histopathology report. Biopsy from the lesion in the CBD showed marked fibrosis with mild to moderate chronic inflammation. IgG/IgG4 Immunohistochemistry was carried out and a diagnosis of an IgG4 related sclerosing disorder was excluded. The pancreatic core biopsy yielded no specific lesion. Peroperative trucut liver biopsy showed features of biliary obstruction.

In view of the failed ERCP, inability to stent the bile duct and the suspicion of IMT, at the second surgery, a debulking of the lesion with a roux-en-Y choledoco-jejunostomy to establish biliary drainage was done. The histopathology of the lesion showed marked fibrosis with presence occasional short fascicles of spindle shaped cells admixed with moderate infiltrates of lymphocytes including occasional small lymphoid aggregates, plasma cells, histiocytes and occasional eosinophils (Fig. 2a and Fig. 2b). On Immunohistochemistry, the spindle cells were positive for smooth muscle actin (Fig. 2c) and showed cytoplasmic positivity for anaplastic lymphoma kinase-1(ALK-1) (Fig. 2d). Thus the diagnosis of IMT was confirmed.

**Figure:**
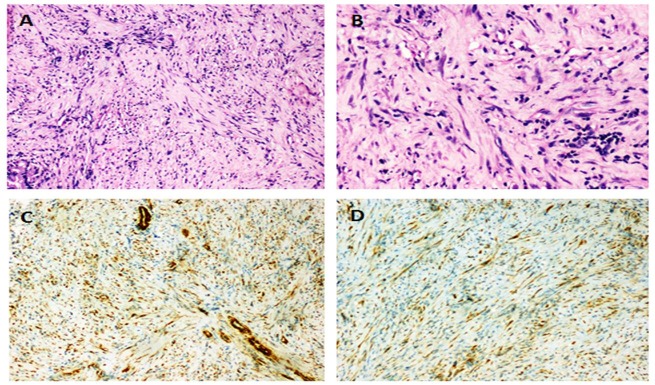
Figure 2:Photomicrographs of the excised lesion; 2a: Spindle shaped cells arranged in fascicles in a background of chronic inflammatory cells (H and E 100x). 2b: Spindle shaped cells in a background of chronic inflammatory cells (H and E 200x). 2c: Spindle cells staining positive for Smooth muscle actin immunohistochemistry (100x). 2d: Spindle cells staining positive for ALK-1 immunohistochemistry (100x).

The postoperative period was uneventful. Prior to removal of the PTBD, a dye study confirmed the free flow of bile into the bowel. She was given a short course of oral steroids followed by a Cox-2 inhibitor (celecoxib). A follow up US at 6 weeks showed a mildly enlarged liver with no ascites, intrahepatic biliary dilatation or any mass. The child has gained weight and is well nine months after surgery. Long term follow-up is planned.

## DISCUSSION

IMTs are true neoplasms with rarely reported metastasis having the distinctive feature on histopathology with the presence of neoplastic spindle cells and an inflammatory infiltrate of plasma cells, eosinophils and lymphocytes.[4] Similar findings were noted in the index case. Additionally, on immunohistochemistry, these spindle cells stain positively for vimentin and smooth muscle actin.[3] The etiology of IMT of the biliary tree in adults is attributed to autoimmune or sclerosing lympho-plasmocytic pancreatitis.[5] However in our case the pancreas did not show any specific lesion or inflammatory/sclerosing process. Moreover, IgG4 which would indicate an ongoing autoimmune inflammation was also negative.

Imaging may not be diagnostic in IMT but contributes to surgical planning if a high index of suspicion is maintained. Fibrous tissue is hypoechoic and relatively hypovascular on US.[6] On MRI, these lesions are hypo to isointense on T1-weighted images and show a marked hypointensity on T2-weighted images. Additionally, they show homogeneous or heterogeneous increased intensity of enhancement on delayed images.[7] An image guided biopsy may further contribute to the diagnosis and surgical decision making. Unfortunately, so far there is no preoperative investigative modality available to rule out malignancy.

Surgery remains the mainstay of management in biliary IMTs for the purpose of relieving biliary obstruction and ruling out a malignant lesion. The importance of intraoperative frozen section biopsies must be stressed to avoid extensive unwarranted resection. In the present case, a staged procedure was performed after the initial biopsy ruled out malignancy. This may be acceptable since it permitted a planned limited resection.

Jun-Jie et al, in a study of intraabdominal inflammatory pseudotumor noted that 25 of 31 cases of IMT resolved completely with conservative measures including use of steroids, antibiotics, anti-inflammatory agents.[8] Recently crizotininb, an oral inhibitor of ALK tyrosine kinase has been introduced as a new therapeutic alternative.[9] A conservative approach may be possible in children if CBD stenting can be achieved. We used a short course of oral prednisolone followed by celecoxib which yielded an impressive clinical and radiological response.

## Footnotes

**Source of Support:** Nil

**Conflict of Interest:** None declared

